# I want to be safe: understanding the main drivers behind vaccination choice throughout the pandemic

**DOI:** 10.1186/s12889-024-18511-z

**Published:** 2024-04-22

**Authors:** Marco Marini, Alessandro Demichelis, Dario Menicagli, Giovanna Mancini, Folco Panizza, Ennio Bilancini, Gustavo Cevolani

**Affiliations:** IMT School for Advanced Studies Lucca, Lucca, Italy

**Keywords:** Vaccine perception, Covid-19, Vaccine hesitancy, Framing effect, Trust

## Abstract

**Background:**

Despite being a major advancement in modern medicine, vaccines face widespread hesitancy and refusal, posing challenges to immunization campaigns. The COVID-19 pandemic accentuated vaccine hesitancy, emphasizing the pivotal role of beliefs in efficacy and safety on vaccine acceptance rates. This study explores the influence of efficacy and safety perceptions on vaccine uptake in Italy during the pandemic.

**Methods:**

We administered a 70-item questionnaire to a representative sample of 600 Italian speakers. Participants were tasked with assessing the perceived effectiveness and safety of each vaccine dose, along with providing reasons influencing their vaccination choices. Additionally, we conducted an experimental manipulation, exploring the effects of four framing messages that emphasized safety and/or efficacy on participants’ willingness to receive a hypothetical fourth vaccine dose. Furthermore, participants were asked about their level of trust in the scientific community and public authorities, as well as their use of different information channels for obtaining COVID-19-related information.

**Results:**

Our study reveals a dynamic shift in vaccine efficacy and safety perceptions throughout the COVID-19 pandemic, potentially influencing vaccination compliance. Initially perceived as more effective than safe, this assessment reversed by the time of the third dose. Beliefs regarding safety, rather than efficacy, played a significant role in anticipating future vaccinations (e.g., the booster dose). Safety-focused messages positively affected vaccination intent, while efficacy-focused messages showed limited impact. We also observed a changing trend in reasons for vaccination, with a decline in infection-related reasons and an increase in social related ones. Furthermore, trust dynamics evolved differently for public authorities and the scientific community.

**Conclusions:**

Vaccine perception is a dynamic process shaped by evolving factors like efficacy and safety perceptions, trust levels, and individual motivations. Our study sheds light on the complex dynamics that underlie the perception of vaccine safety and efficacy, and their impact on willingness to vaccinate. We discuss these results in light of bounded rationality, loss aversion and classic utility theory.

## Introduction

**Fig. 1:**
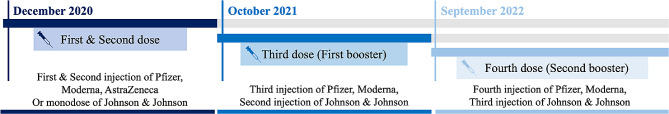
Covid-19 Vaccine Doses and Scheduling in Italy. The figure illustrates the different doses of the vaccine along with their scheduling. Each colored line represents a different dose schedule, with corresponding labels indicating the number of doses and the time intervals between them

In March 2020, the World Health Organization declared Sars-Cov-2 a pandemic [1]. To immediately address the high number of hospitalizations and deaths, institutions worldwide implemented Non-Pharmaceutical Public Health Interventions (NPHIs), such as stay-at-home orders, social distancing, travel restrictions, and lockdowns [2–4]. At the same time, some pharmaceutical companies pursued the development of vaccines against COVID-19 [5]. The BioNTech/Pfizer, Moderna and AstraZeneca vaccines received approval from the European Medicines Agency (EMA) in December 2020 [1]. Afterward, the vaccination campaign in all EU member states began on 27 December 2020 [6]. The first vaccination cycle (the first and second dose) included two doses administered a few weeks apart (usually 3–12 weeks; [7]). As the pandemic progressed and new virus variants emerged, most European countries responded by implementing additional booster doses [8] to combat the rising epidemiological curve (Fig. 1).

Despite the overwhelming evidence demonstrating the public health benefits of vaccines, vaccine hesitancy has become an increasingly pressing concern on a global scale [9]. The WHO defines vaccine hesitancy as the “[…] delay in acceptance or refusal of vaccines despite availability of vaccination services.” (WHO, 2021). Vaccine hesitancy is a multi-faceted phenomenon, which raises a series of ethical, social, epistemic, and psychological challenges [10]. It was an issue present even before the pandemic, but with COVID-19 it came to the fore worldwide. Specific attitudes towards COVID-19 vaccination have been influenced by several factors: pre-existing opinions towards vaccination [11], perceived risks and benefits associated with the vaccine [11], trust in authorities and scientific community [12], acquaintances’ opinion [13], common doubts about vaccine development and approval process [14], and socioeconomic factors [15].

For all the above reasons, understanding vaccine hesitancy and its determinants is not an easy task. However, it is now clear that the perception of the safety and efficacy of the vaccines is an essential factor influencing vaccination choice, and that concerns about the vaccine’s safety and efficacy are among the main drivers of vaccination refusal or delay [16, 17]. Accordingly, the present cross-sectional study focused on COVID-19 vaccine safety and efficacy perception and tried to assess their influence on the acceptance of vaccines in Italy during the pandemic. A preregistered survey investigated safety and efficacy perceptions of COVID-19 vaccine doses among 600 Italian speakers. Participants were also randomly assigned to read one of four messages to understand how efficacy and safety perception influenced their willingness to receive a new dose. In addition, we retrospectively investigated how internal and external reasons shaped vaccination decisions during the pandemic and how trust in public authorities and scientific communities evolved as crucial factors modulating vaccine perception.

## Understanding willingness to vaccinate in the COVID-19 pandemics

Recent research has increasingly illuminated the reasons and determinants of vaccination choices [18, 19]. The resulting picture is a nuanced one, highlighting a number of different factors both promoting and hampering willingness to vaccinate. In this section, we briefly discuss the most important factors affecting the vaccination campaign against COVID-19, as motivating our choices in designing the survey. The COVID-19 pandemic represented an extraordinary event due its global diffusion and prolonged duration. The active involvement of the world population in the prevention and mitigation of this emergency, along with the roles played by international and national health systems, highlighted the fundamental relevance of social context and psychological drivers in ensuring an efficient understanding and response to face this type of phenomenon. During the period of our study, Italy had already administered the first booster dose of the vaccine to a significant portion of the population (68%; https://www.salute.gov.it) and had initiated the administration of the second booster dose, primarily targeting elderly and vulnerable individuals. At that time, Italy was contending with the emergence of evolving variants of the virus, (i.e., the Omicron variant), which underscored the urgency of progress in the vaccination campaign. While traditional and social media consistently emphasized the importance and necessity of the second booster doses, the Ministry of Health recommended vaccination for individuals over 60 or those considered frail, yet lacked a definitive mandatory directive (https://www.salute.gov.it). Media coverage and public discourse remained heavily focused on vaccination efficacy and the imperative of achieving widespread immunity to mitigate the virus’s transmission and potential impact on public health and the economy.

### Perception of the safety and efficacy of the vaccines

Starting with our main focus, efficacy and safety perception is one of the main drivers of vaccination choice [20–22]. During the pandemic many studies proved that perceived risk and concerns regarding the safety and efficacy of the vaccines had been the primary factors that influenced the acceptance of new vaccines (for a review, see [23]). Recent findings suggested that the decline in vaccination intentions may be linked to both exposure to COVID-19 misinformation and public concerns about vaccine safety [24]. Similarly, concerns about vaccine safety represented the main reasons behind vaccine refusal [25, 26]. These concerns appear to be rooted in the rapid development of COVID-19 vaccines, limited information available, and anxieties about mild and transient side effects. Conversely, other studies propose that the perception of efficacy plays a crucial role in the decision to accept a vaccine [27, 28]. The pivotal role of these two concepts has been widely recognized. However, very little is known about the diachronic evolution of citizens’ efficacy and safety perceptions and how these factors have changed over time, affecting vaccine uptake rates. To date, several studies have shown that vaccine compliance varied depending on the stage of the pandemic [29]. However, a first measurement problem is related to the time when efficacy and safety perceptions were recorded (i.e., before or after the vaccine launch). Specifically, variations in individuals’ willingness to receive the vaccine may be influenced by the timing of their perceptions. Indeed, during the initial peak phase of the pandemic, individuals may have been more willing to receive the vaccine. Nevertheless, once the vaccine was released, their concern about its safety could have changed their attitude [22]. Secondly, it has been shown that fluctuation in vaccine efficacy perception was associated with a considerable variation in the level of acceptance of COVID-19 vaccines [30]. For example, confidence in vaccine efficacy (and not safety) was also found to be the main predictor of vaccine intention among Italian nurses (32; for an opposite result, see 0). To the best of our knowledge, there is no clear trend regarding the influence of safety and efficacy perception on the willingness to vaccinate. For example, confidence in vaccine safety and efficacy has improved over time [33] but after the release of the booster doses, safety (mis)perception was still the main driver for vaccination hesitancy [33]. Worryingly, a study by Wang and colleagues [30] reported a general decrease in vaccine perceived safety during the third wave conjoined with a decrease in the willingness to vaccinate. Similarly, the protracted debates surrounding vaccination have significantly impacted people’s overall attitudes toward vaccines, resulting in increased skepticism regarding their efficacy [35]. Nevertheless, given how the COVID-19 vaccine development was rushed due to the exceptional circumstances, the perception of insufficient testing undermined confidence in their efficacy and safety [36]. These fluctuations in vaccine perceptions may offer insights into understanding changes in vaccination rates over time. These findings highlight the complex and evolving nature of vaccine attitudes and underscore the need for ongoing research not to merely be satisfied with an analysis of the factors influencing vaccine decision-making, but also keep in mind the temporal evolution of them. To comprehensively analyze such expected discrepancies at different points in time, we investigated how vaccine safety and efficacy perception has evolved among different doses. In our cross-sectional study, participants were asked to report their perceived safety and efficacy of the vaccine at the time of each dose administration. Therefore, our study is aimed at building on and expanding those previous works by trying to understand how the perception of efficacy and safety changed during the vaccination campaign.

### Framing safety and efficacy and its impact on willingness to vaccinate

To date, numerous behavioral interventions have been implemented to mitigate vaccine hesitancy [37]. Some of the most used behavioural interventions are those that seek to modify the context in which decisions are taken. This “choice architecture” has been modified in several ways, such as the use of (non-) monetary incentives [38], default options [39], manipulation of the type and source of information (i.e., the protection against diseases, the potential to save lives, the positive impact it can have on public health, and the safety of the vaccine [25, 40]). Several types of manipulation are built using the so-called Prospect Theory. First proposed by Kahneman and Tversky [41], this theory posits that individuals, when facing a decision with outcomes that can be translated in gains and losses, treat those two outcomes in an asymmetrical way. Averting a loss is, on average, a stronger motivator than seeking a gain. This implies that the same information, presented (“framed”) in a format that emphasizes loss or gain, could have different impacts on vaccine uptake. COVID-19 vaccination campaigns were not different in that regard [42–45]. Loss-framed information, which emphasizes the probability of contracting the virus, proved to be more effective than gain-framed messages in increasing people’s risk perception [46–49], in most, even if not every, study [50]. Keeping the implication of prospect theory in the background allows to disentangle the impact of two elements that are often considered together in studies on vaccine hesitancy: efficacy and safety. However, efficacy can be considered a measure of how much benefit one can gain from taking a vaccine, and safety is a measure of how little harm is carried by the decision to vaccinate. Of the several interventions considered in the literature, to the best of our knowledge, no study tested the effect of messages that focus on just the safety or the efficacy of the vaccine and the present study aims at filling this gap.

### Internal and external reasons behind vaccination choice

When studying the decision to get vaccinated it is important to consider the intrinsic motivations that drive an individual choice [51] and can modulate the safety and efficacy perception of the vaccine [52]. These motivations can be broadly classified into two categories: inner and outer factors [53, 54]. Inner factors are based on self-regarding reasons, such as wanting to protect oneself from the virus and reduce the risk of contracting COVID-19. Outer factors are more social and other-regarding in nature, for example, wishing to protect others from the virus by reducing the spread of COVID-19 [55]. Additionally, individuals may feel pressure from their social networks to receive the vaccine [56]. People attitudes and behavior towards vaccine uptake are also influenced by their perception of social norms, particularly those derived from close relationships. For example, the favorable behaviors of friends and family members predicted an alignment with their choices [58, 59]. Similarly, the expectation of the general vaccination rate among others was found to impacted people willingness to vaccinate [13]. In summary, social norms play a crucial role in determining an individual’s decision to vaccinate, and peer influence can amplify this effect [59].

### Trust, and its epistemic and intrinsic consequences

Trust is relevant because it represents a crucial factor in vaccine compliance [60]. It enables individuals to evaluate the safety and efficacy of the vaccine, based on the reliability and trustworthiness of both the scientific community that produced the vaccine, and the institutional authorities that oversee vaccination campaigns [61, 62]. Furthermore, in the context of COVID-19, the rapid development of vaccines and the rigorous measures adopted have further eroded people’s trust [63]. However, it should be kept in mind that trust in institutions and trust in the scientific community have different traits, and influence people in different ways. On one hand, public authorities are expected to be trusted because of their role in serving and protecting individuals. As shown by Sapienza and Falcone [64] trust in public authorities is in turn shaped by the complex relationship between the trustor and trustee in which the former imposes measures on the latter. On the other hand, the scientific community is expected to be trusted because of their specialized knowledge and expertise in their field [65]. Indeed, trust in the scientific community can be seen as a robust predictor of vaccine compliance, and it should be prioritized once a vaccine becomes available [66]. Moreover, the process of building and losing trust is dynamic and ever-changing. While at the beginning of the pandemic there was a general “trust boom” towards both public authorities and scientific community, a correspondingly rapid decrease was observed with the evolving information on vaccines and changing government policies during the pandemic [67, 68].

### The present study

Our intention was to provide an overview of Italy’s fourth dose COVID-19 vaccination intention rate, as of May 2022. Accordingly, we measured the time-sensitivity of crucial factors on the willingness to vaccinate during the vaccination campaign, as well as the safety and efficacy vaccine perception, the trust in the scientific community and government, the self- and other-regarding reasons and the social norms leading to the vaccination decision.

Given that the number of infected individuals did not substantially decrease over time, despite a gradual increase in the number of vaccinated people, we expected a decline in the vaccine’s perceived efficacy over time while anticipating an increase in safety perception. Additionally, drawing upon the principles of prospect theory [41], wherein the fear of facing adverse side effects may be a more compelling motivation than the achievement of global immunity, we hypothesized that messages highlighting the absence of risks would result in a greater willingness to receive the fourth dose compared to messages emphasizing vaccination benefits [69]. As vaccine effectiveness perception declined, we also expected a decrease in COVID-19-related concerns but an increase in perceived social pressure. Lastly, our investigation into trust evolution aligned with prior research [70] which suggested a significant decline in trust towards institutional authorities compared to the scientific community, mainly due to the increasing reservations about how institutions have managed the crisis in Europe.

## Methods

### Participants

We conducted an online survey on Qualtrics.com between October 25 and October 31, 2022, with a stratified sample of Italian native speakers (*N* = 600). The present study design was preregistered prior to beginning data collection (https://osf.io/k3dt2). Participants were balanced on age clusters, sex (F = 248), educational level (Middle school or lower; High School Diploma; Bachelor’s degree or higher), and region of recruitment (Northern, Central, and Southern Italy). The main sociodemographic characteristics of the sample are summarized in Table 1. At the moment of data collection, many European countries had just started administering the IV dose (or second booster dose). In Italy, 84% of the population was vaccinated, and 68% received both the first and third doses (i.e., they were fully vaccinated). A pilot study was previously conducted (*N* = 90) to provide a general indication for developing an accurate experimental design. Participants for the present study were recruited via an incentivized online panel specialized in data collection in Europe (Bilendi.com). After data screening, 108 (18%) participants were excluded from the sample according to the following exclusion criteria: (a) failure to provide informed consent to participate (1 participant); (b) incompletion of the survey (pre-registered; 10 participants); (c) time for completion (6 participants took too long and 62 participants took too short to conclude the survey; < 210 s for the short version of the survey or 300 s in the full-length version; timing threshold followed pilot indications and experimenter trials. This criterion was not included in the preregistration, but it became necessary due to the numerous expedited responses received); e) repeated responses across trials (preregistered; 22 subjects selected the same response for > 85% of the items in more than 1 Likert scale (with reversed items) suggesting inattentive responses; f) incoherent answers in following questions about the vaccination outcome (preregistered; 7 participants). The present research obtained ethical approval from the ethical committee of the University of Siena (16/2021) and was developed following the American Psychological Association guidelines for behavioral ethical research.

**Table 1:** Sample characteristics

	Male % (49%)	Female % (50%)	Total %
**Age**			
18–30	15.8	16.7	16.3
31–40	22.1	21.5	21.8
41–50	21.3	20.3	20.8
51–60	19.6	19.9	19.8
+ 60	21.3	21.5	21.4
**Level of education**			
Middle school or lower	22.7	28.2	25.5
High School Diploma	36.0	40.3	38.2
Bachelor’s degree or higher	41.3	31.5	36.4
**Geographical distribution**			
Northern Italy	36.5	28.7	32.6
Central Italy	31.5	36.8	34.2
Southern Italy/islands	32.0	34.4	33.2
**Covid contagion**			
Yes	52.9	50.2	51.6
No	47.1	49.8	48.5
**Green pass Certificate**			
Yes	84.6	79.4	82.0
No	15.4	20.6	18.0
**Number of doses**			
0	14.0	18.5	16.3
2	11.2	12.5	11.9
3	74.8	69.0	71.9

### Hypotheses

The current research project investigated the citizen perception of the vaccine during the pandemic with four main preregistered research questions (see Table 2):

1. **Evolution of the perceived safety and efficacy**. Our first aim was to explore the diachronic evolution of the vaccine’s perceived safety and efficacy regarding the previously administered doses (I and II; III). Specifically, we anticipated a decline in the perception of vaccine efficacy over time as the rise in the number of vaccinated individuals did not align with the trend of infection cases. That is, the fact that the number of infected people did not substantially reduce over time would have cast doubts about the vaccine’s efficacy. However, we assumed that the gradual increase in the number of vaccinated people and the choice to continuously adhere to the vaccination plan was mainly supported by an increased perception of vaccine safety (preregistered RQ1).
2. **Framing effect.** In addition, our experimental manipulation aimed to examine how various messages impact the willingness to adhere to the vaccination schedule, specifically by receiving the fourth (II booster) dose administration. Building on the principles of gain-loss asymmetry and prospect theory [41], we hypothesized that messages emphasizing the absence of vaccination-related risks (Safety condition) would lead respondents to be more inclined to receive the fourth dose compared to messages that focused on the potential benefits of the vaccination (Efficacy condition) (preregistered H1).
3. **Reasons behind vaccination**. Secondly, we aimed to investigate how the reasons for choosing to get vaccinated evolved during the pandemic. Specifically, we hypothesized that as the perception of vaccine effectiveness declined, the motivations for getting vaccinated due to COVID-19-related concerns would also decrease. However, we anticipated that the introduction of a mandatory GP certificate during the third dose administration period for engaging in work and social activities would increase the perceived social pressure to get vaccinated (H2).
4. **Trust evolution.** Lastly, we explored the diachronic evolution of trust among public authorities and the scientific community. In line with previous studies [71], we hypothesized a greater decrease in the trust in vaccine guarantors compared to the trust in vaccine producers (preregistered RQ2).

**Table 2:** Main research questions

Research questions	Variables
1. Change in the safety and efficacy perception between different doses (preregistered research question)	Efficacy in I and III Safety in I and III
2. The effect of the framing on the willingness to vaccinate (preregistered hypothesis)	4 b-subjects conditions (EC; SF; ESC; CC) Vaccination intention & availability
3. Diachronic evolution of personal motivation behind vaccination choice	Covid-related & social pressure reasons in I and III
4. Diachronic change in trust attribution among producers and guarantees (preregistered research question)	Trust in the scientific community & public authorities

### Survey structure and measures

**Fig. 2:**
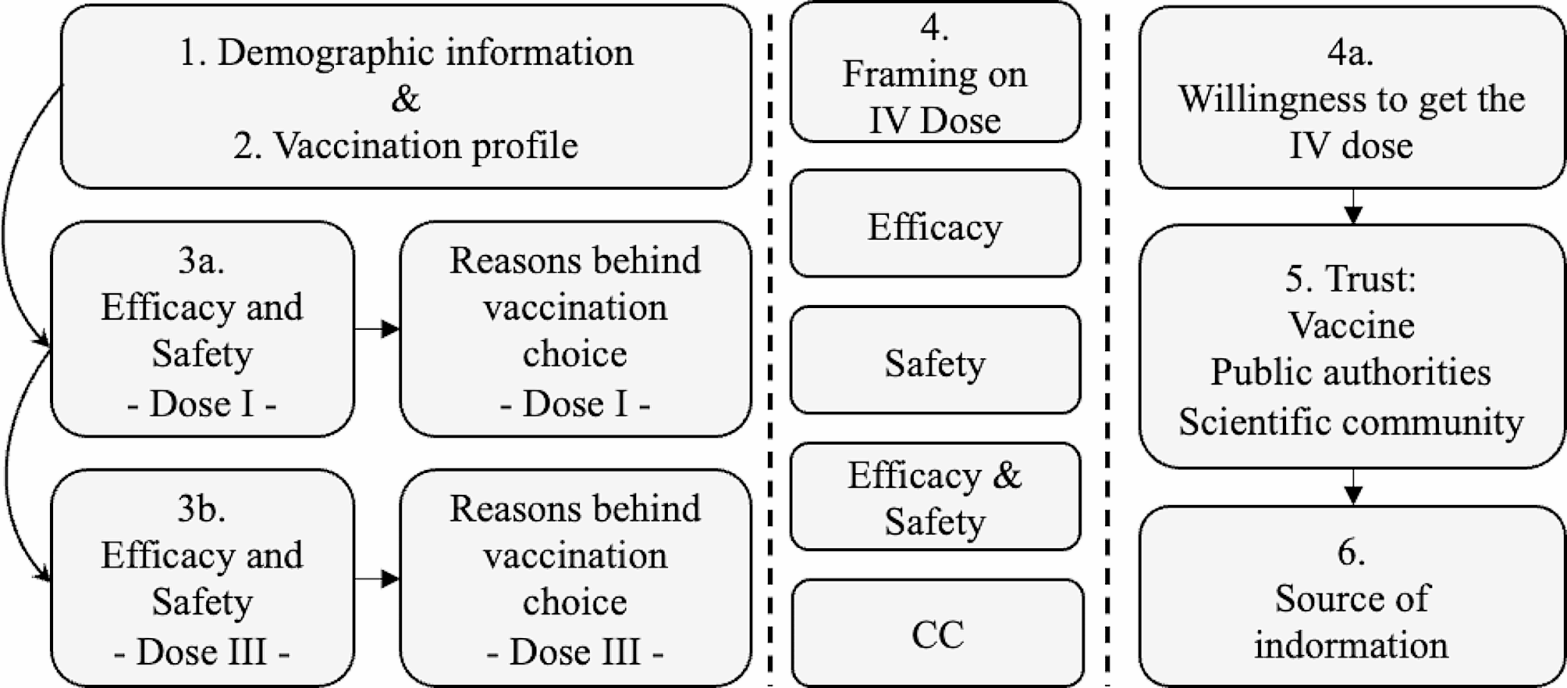
Survey structure and experimental conditions

An anonymous, 70-item preregistered questionnaire was developed and hosted on Qualtrics, a website that collects survey responses online. The average duration of the survey was 10 min (*SD* = 9.35 m). Participants could reach the survey from their laptops or smartphones via a link valid for a single use. The first page of the questionnaire provided a general overview of the study, its main objectives, and its instructions. Participants were asked to provide their consent to participate before proceeding. The questionnaire was divided into the following six main sections (Fig. 2):

1. Demographic information: the main socio-demographic data were collected at the beginning of the survey. Participants answered questions about age, gender, geographical origin, education level, employment type, annual income, and flu vaccination.
2. Covid-related information: All participants were profiled to evaluate their compliance with the vaccination campaign. Specifically, they answered questions about the number of received COVID-19 vaccine doses (Fig. 1), whether they had contracted the virus, and if they had obtained the European Green pass (GP) certificate. GP was a digital document mandatory from December 2021 to December 2022 to carry out work and social activities, issued by the Ministry of Health, attesting compliance with the vaccination plan.
3. Vaccine perception and reasons behind vaccination: depending on the participant profile, each respondent answered two questions about the perceived safety and efficacy of the vaccine at the time of each dose administration (I and III doses; i.e., “When I received the first/third dose of the COVID-19 vaccine, I thought the vaccine was safe/effective”) using a 4-point Likert scale (from “strongly disagree” to “strongly agree”). Subsequently, five items investigated the reasons behind the vaccination choice for each dose (I and III) in terms of (a) contagion-related reasons (“I did not want to get the virus”; “I did not want to infect other people” I dose α = 0.86; III dose α = 0.80); (b) social (pressure) reasons (“I did not want to be judged by others”; “My acquaintances had been vaccinated”; “My colleagues had been vaccinated”; I dose α = 0.80; III dose α = 0.82).
4. Information conditions regarding safety and efficacy of the IV (or II booster) dose: before expressing the willingness to get the fourth dose of the vaccine, participants who received the three previous doses were randomly assigned to one of four experimental conditions in which they read different information about the last vaccine dose available:

- Efficacy condition (EC): In EC, subjects were informed that “The latest data available to the scientific community that have been published in the New England Journal of Medicine, one of the most authoritative international scientific journals, highlighted the high Effectiveness of the Fourth Dose in protecting against the contagion from COVID-19”. This condition highlighted the efficacy of the fourth dose.
- Safety condition (SC): SC specified that “The latest data available to the scientific community that have been published in the New England Journal of Medicine, one of the most authoritative international scientific journals, highlighted the high Safety of the Fourth Dose concerning the onset of side effects”. This condition highlighted the safety of the fourth dose.
- Efficacy and safety condition (ESC): ESC reported both previous information (EC; SC) in a counterbalanced order.
- Control condition (CC): CC specified that the research community has already collected the efficacy and safety data about the fourth dose, and they will soon be available also to the non-scientific community.

Immediately after the framing (i.e., information conditions), participants indicated their intention to get the fourth (or second booster) dose of the vaccine through two consecutive questions: a) using a 4-point Likert scale (from “strongly disagree” to “strongly agree”) to answer the question “I will take the fourth dose of the vaccine as soon as possible”; this question provided a direct response regarding participants’ willingness to take the fourth dose. b) specifying, using an ordinal 7-point scale, “In which case would they be willing to get the fourth dose?” (from “I’ve already got it” to” I am not willing to get the fourth dose”); this question explored the conditions under which participants might consider getting the fourth dose.

5. Trust assessment: within this section, participants responded to two different Likert scales on their trust in (a) the scientific community (ScC) and (b) public authorities (PA). The two scales evaluated participants’ (i) general trust in terms of competence, reliability, intention, (PA α = 0.89; ScC α = 0.88); and (ii) COVID-19 related trust encompassing aspects such as vaccine overestimation, corruption, and loss of trust (PA α = 0.88; ScC α = 0.88). The two scales were adapted from a previous study [67] by adding two items (“Vaccines are not as effective as the public authorities/scientific community claimed at an early stage of the pandemic”; “I progressively lost trust in the public authorities/scientific community over the course of the pandemic”) measuring the change in trust attribution during the pandemic. Subsequently, we explored the participants’ opinions on vaccines in general through a 10-item scale (α = 0.92; aVHS; [72]). The tree scales of this section were presented in a randomized order.
6. Source of information: this last section investigated the frequency of use of 8 different sources of information channels: Acquaintances, colleagues, traditional media, social networks, the internet (YouTube and blogs), the scientific community, and government channels.

### Statistical analyses

IBM SPSS 26 [73] was used for main statistical analysis. The effect sizes were provided by using the η value (eta squared for ANOVA statistics). We set the significance to *α* = 0.05. All variables were tested for normality by the Shapiro–Wilk test and homoscedasticity by the Levene test. In case of violation of parametric analysis assumptions, the Z score for large samples was computed by referring to the skewness and kurtosis measures for determining the normality distribution [74]. All the ANOVA post-hoc comparisons were corrected using the Bonferroni correction and were investigated upon a significant main effect or interaction. The path analysis reported the path coefficient in the standardized form (β), the relative Standard Error (S.E.), the statistical significance (*p* value), and a 95% Confidence interval (95% CI) for each path.

## Results

Once data collection was completed, we verified that our sample could be comparable with the general Italian population as regards vaccination rates. Most of our sample (84%) received at least one dose of the vaccine (vs. 84% of the Italian population), and 72% completed the vaccination course (I, II, and III doses; vs. 68% of the Italian population, as reported by www.governo.it at the time of data collection). Moreover, 51% of our respondents reported having contracted the virus as compared to the 40% of the Italian population. As for the demographic characteristics, the study sample was balanced by gender, age, level of education, and geographical provenience (see Table 1).

**Fig. 3:**
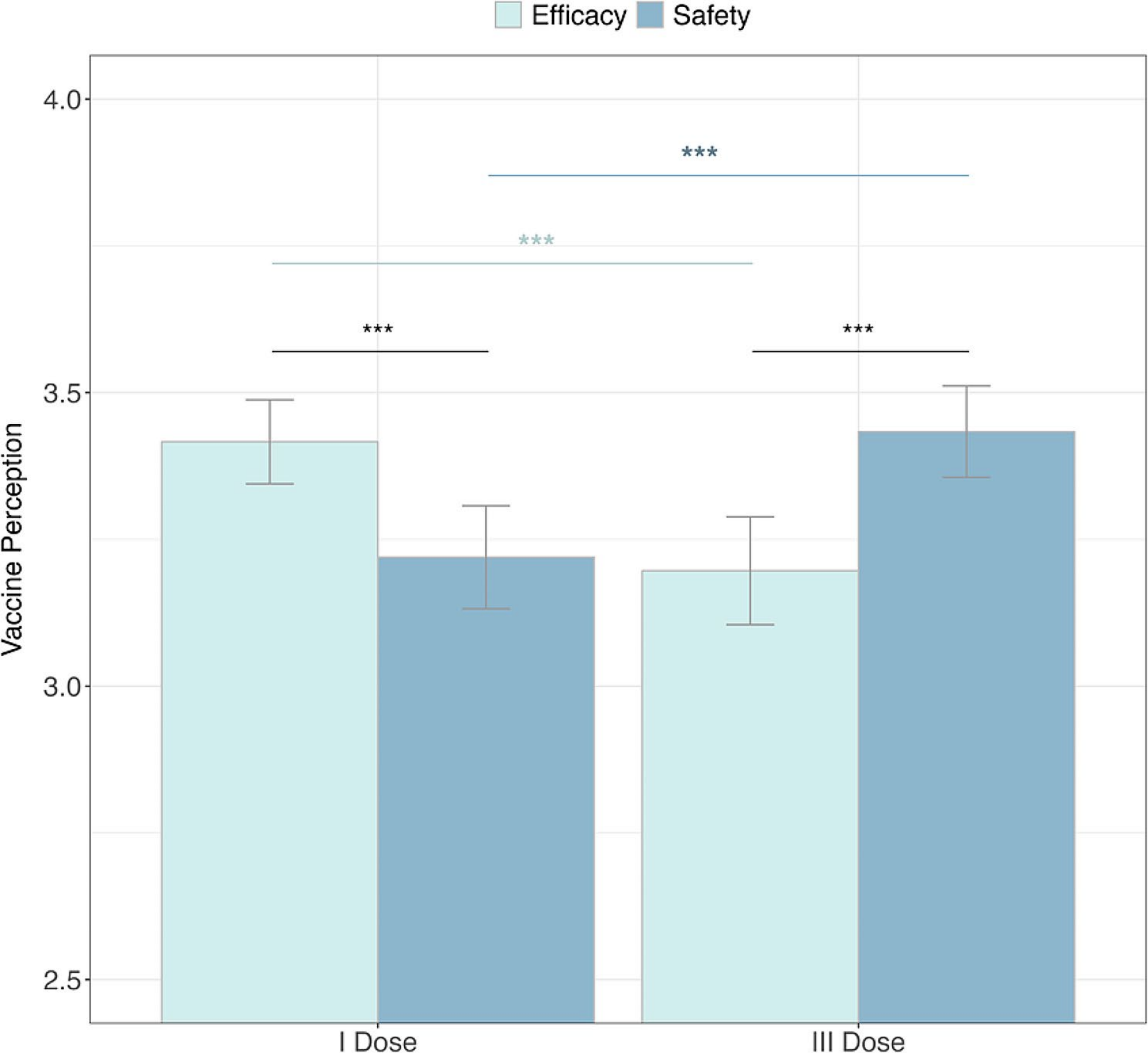
Diachronic evolution of safety and efficacy of the vaccine between the first and third dose administration period. The progression of the pandemic has led to a decrease in the perception of vaccine efficacy and an increase in the perception of its safety. Error bars indicate 95% confidence intervals; ***p <.001

Our first research question (RQ1) aimed to explore the diachronic evolution of the perceived vaccine characteristics during the pandemic. A two-way repeated measure ANOVA 2 perceived vaccine characteristics (recalled Efficacy and recalled Safety) × 2-time intervals (I and II doses; III dose) was conducted on participants’ vaccine perception (dependent variable). The ANOVA did not reveal a main effect of the vaccine characteristics (*p* =.40) or the doses (*p* =.91). However, as hypothesized, we found a robust significant interaction between factors (*F* (1, 345) = 61.14, *p* <.001, η_p_^2^ = 0.15, (1 − *β*) > 0.99). Interaction post hoc analysis confirmed a significant drop in the efficacy perception between the first (*M* = 3.42; *SD* = 0.68) and third dose (*M* = 3.20; *SD* = 0.87; *p* <.001) and a significant increase in the perceived safety between the first (*M* = 3.22; *SD* = 0.83) and third dose (*M* = 3.42; *SD* = 0.74; *p* <.001). In summary, during the first dose administration, the vaccine was perceived to be more effective than safe. However, during the third dose administration, it was considered to be more safe than effective (Fig. 3).

**Fig. 4:**
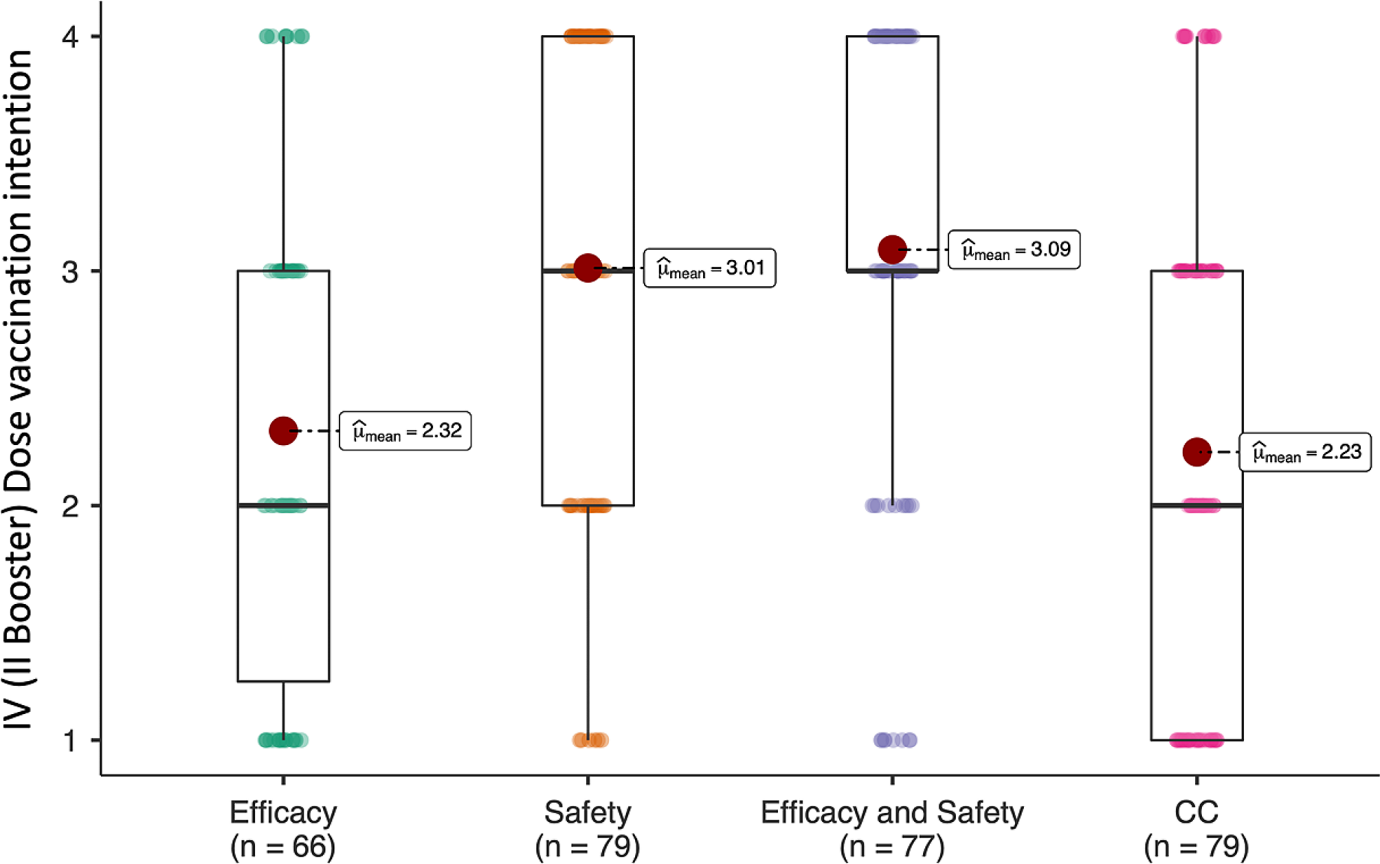
Effect of different messages on the willingness to vaccinate. Participants only showed a greater intention to vaccinate when exposed to messages that contained information about the safety of the vaccine. participants in CC and Efficacy conditions demonstrated comparable vaccination intentions (*p* >.99). However, both groups exhibited lower vaccination intentions compared to participants in the Safety (ps < 0.001) and Efficacy and Safety (ps < 0.001) conditions. In the boxplot, the lower and upper fences correspond to the 25th and 75th percentiles, respectively, with the median positioned in between. The bars depict the 10th and 90th percentiles, while red dots indicate mean values. Colored dots represent individual data points

In order to explore H1 (the different impact of message framing on the willingness to receive the fourth dose), we had to exclude participants who had already got the dose and respondents who did not complete the previous vaccination plan. Subsequently, a one-way ANOVA was conducted to analyze participants’ willingness to get the fourth dose of the vaccine (dependent variable) in the EC (*N* = 66), SC (*N* = 79), ESC (*N* = 77), and CC (*N* = 79) conditions. The results showed a main effect of the condition (*F* (3, 300) = 15.59, *p* <.001, η_p_^2^ = 0.14, (1 − *β*) > 0.99), suggesting an impact of the framing on the vaccination intention. Indeed, post-hoc analysis showed a significant difference between the safety (SC: *M* = 3.01; *SD* = 0.97) and efficacy and safety (ESC: *M* = 3.09; *SD* = 0.98) group vs. the efficacy (EC: *M* = 2.32; *SD* = 1.01) and the control group (CC: *M* = 2.23; *SD* = 1.04; *ps* < 0.001). Moreover, no significant differences were found between EC and CC (*p* >.99) and between SC and ESC (*p* >.99). In a nutshell, messages that highlighted the high safety of the vaccine (ES; ESC) were associated with a greater propensity to take the fourth dose. Conversely, the message that included only efficacy-related information did not increase vaccination intention (Fig. 4).

Subsequently, to investigate the evolution of the COVID-19-related and social pressure reasons during the pandemic (H2), we performed a two-way repeated measure ANOVA 2 reasons (contagion-related reasons; social (pressure) reasons) × 2 doses on the latent variables scores we obtained averaging single reason items. The ANOVA did not reveal a significant main effect of the doses (*p* =.42). Still, it highlighted a significant main effect of the reasons (*F* (1, 345) = 645.24, *p* <.001, η_p_^2^ = 0.65, (1 − β) > 0.99) and a significant interaction *F* (1, 345) = 17.02, *p* <.001, η_p_^2^ = 0.05, (1 − *β*) = 0.98. More specifically, Bonferroni corrected post-hoc comparisons proved a significant decrease in COVID-19 related reasons (CrR) between the first (*M* = 3.42; *SD* = 0.79) and the third dose (*M* = 3.31 *SD* = 0.86; *p* <.001) and a significant increase of the social pressure reasons (SpR) (I Dose: *M* = 1.96; *SD* = 0.87; III Dose: *M* = 2.05; *SD* = 0.95). Namely, with the progress of the pandemic, people chose to get vaccinated less for COVID-19 related purposes and more for social pressure reasons.

Secondly, we performed a path analysis with robust standard errors on 347 respondents with at least III doses to verify if this pattern was related to a general change in the safety and efficacy perception. In greater detail, we computed the difference in the COVID-19 related reasons (CrR at the third dose– CrR at the first dose) and in the social pressure reasons (SpR at the third dose– SpR at the first dose) as the dependent variables and the change in the efficacy and safety perception (Efficacy/Safety at the third dose– Efficacy/Safety at the first dose) being the explanatory variables (IV). Our model highlighted an opposite influence of the efficacy change in building future vaccine motivation. In particular, the decrease in the efficacy perception was positively related to a decrease in COVID-19 related reasons (*β* = 0.21, 95% *CI* [0.13, 0.32], *p* =.03). On the contrary, a decrease in the efficacy perception predicted an increase in social pressure among the doses (*β* = 0.33, 95% *CI* [0.36, 0.44], *p* <.001). Simultaneously, a decrease in the safety perception was associated with an increase in COVID-19 related reasons (*β* = −0.19, 95% *CI* [− 0.32, − 0.03], *p* =.02) and a decrease in social pressure factors (β = 0.27, 95% *CI* [0.09, 0.33], *p* <.001).

Lastly, to examine the different trust among public authorities (PA) and the scientific community (ScC; RQ2), we conducted a preliminary two-way repeated measure ANOVA 2 trustees (PA and ScC) × 2 trust types (General turst and COVID-19 related trust) that reported a main effect of the trustees (*F* (1, 462) = 112.21, *p* <.001, η_p_^2^ = 0.20, (1 − *β*) > 0.99) and a main effect of the trust type (*F* (1, 462) = 155.30, *p* <.001, η_p_^2^ = 0.25, (1 − *β*) > 0.99). In general, PA were deemed as less trustworthy than ScC (*MD* = − 0.24; *p* <.001), and general trust recorded higher values than COVID-19 related trust (*MD* = 0.49; *p* <.001). A fine-grained repeated measure ANOVA we ran on the scores of all the items reported that, concerning participants’ perceptions of COVID-19 related trust, PA and the ScC had similarly overestimated the vaccine efficacy (*p* =.18) and were comparably involved in the vaccine turnover (*p* =.35). However, what is particularly relevant, is that the participants’ trust in PA decreased significantly more as compared to the ScC trust during the pandemic (*MD* = − 0.35; *p* <.001).

## Discussion

The study extensively explored vaccine hesitancy, with a particular emphasis on elucidating the dynamics associated with the perceived safety and efficacy of the vaccine amid the backdrop of the COVID-19 pandemic. Our study investigated the diachronic evolution of the perception regarding vaccine characteristics, revealing a noteworthy shift in recalled perceptions of efficacy and safety across different doses. Specifically, our subjects recalled a decline in efficacy perception and a simultaneous increase in safety perception when they referred back to their past vaccination decisions. Building up on that element, our findings underscored the role of messages emphasizing heightened vaccine safety in increasing individuals’ willingness to vaccinate as compared to those focusing on a high vaccine efficacy, which failed to elicit a similar effect. Additionally, an investigation into the temporal evolution of COVID-19-related and social pressure reasons revealed a significant decrease in COVID-19-related motivations alongside a parallel increase in motivations influenced by social pressure. Lastly, the examination of trust in public authorities (PA) and the scientific community (ScC) disclosed not only a perception of PA as less trustworthy than ScC but also a notable decrease in trust in PA compared to trust in ScC over the course of the pandemic. These findings contribute valuable insights into the intricate interplay of vaccine perceptions, messaging, motivations, and trust dynamics within the continually evolving context of the COVID-19 pandemic.

### Perception of the safety and efficacy of the vaccines

Within the vaccine hesitancy debate, concerns about the safety and efficacy of vaccines proved to be some of the most prominent drivers of vaccine refusal or delay [19]. In our study we retrospectively investigated the diachronic evolution of the vaccine’s perceived safety and efficacy throughout the pandemic. Our findings showed that if the vaccine perceived efficacy decreased as the pandemic progressed, the increase in the number of administered doses was associated with an increased perception of vaccine safety. What are we asking is: why? Our data are compatible with an explanation that can be modeled with classic utility theory [75]. At the beginning of the pandemic, the risk carried by catching COVID-19 was high [76]. Reports of hospitalization, suffering, and death made the disease a terrifying perspective for many [77]. In this context, the efficacy of a new vaccine was an extremely salient feature, given how high were the costs of catching the disease. Safety, on the contrary, was less salient (even if still quite relevant), given how the opportunity costs of not getting vaccinated could trump the danger of incurring in Adverse drug reactions(ADR). However, as the pandemic unfolded, this situation evolved. The key factor here is that vaccination was carried out on several different doses [78].

For each dose received, the perception of importance of subsequent doses (or, if you prefer, their marginal utility) was reduced [79, 80]. A clarification with fictional numbers: if one person thinks that with the first dose she would obtain protection by a factor of 50, with the second dose by 75, and with the third dose by 90, it would mean that, roughly, the correspondent perceived importance of each dose would halve for each following inoculation. Moreover, the rising occurrences of increasingly common harmless reinfections, often with mild symptoms, may have negatively influenced individuals’ perceived necessity for additional vaccine doses. Safety, on the other hand, would see its importance increased with each dose, because the probability of ADR could be seen as increasing and cumulative with each subsequent dose. Even if it is not seen as cumulative, the associated potential of ADR that can be considered acceptable for a live-saving vaccine can be deemed unacceptable for a dose that covers a situation deemed not anymore life-threatening. Additionally, the positive experiences reported by individuals and their acquaintances with each vaccine dose might have contributed to the reassuring perception of safety over the long term. The accumulation of mostly positive experiences could have played a role in shaping a favorable view of vaccine safety.

### Framing safety and efficacy and its impact on willingness to vaccinate

The purely experimental section of our study explored the role of efficacy and safety in shaping willingness of future vaccination. The effect we observed was that the influence of a communication based on the efficacy was negligible, whereas safety played an impactful role. Among the vaccine features we presented, the primary concern of potential vaccine candidates turned out to be its safety. Our results showed that providing positive information on the next dose’s safety (i.e., the small number of side effects and their low relevance) significantly increased people’s willingness to vaccinate compared to messages emphasizing the new dose’s excellent efficacy (in preventing contagion). What is even more interesting is that messages that reported both efficacy and safety information did not show a higher willingness to vaccinate compared to messages that included only safety information. Spinning vaccine efficacy in a positive light did not affect vaccination compliance; The only thing that mattered was the vaccine’s safety. In short, those who expressed their willingness to get vaccinated increasingly cared just to be safe.

An explanation of this effect may be provided in terms of prospect theory [41, 81]. Specifically, it underlines the loss-gain asymmetry, which states that there is a cognitive tendency to express a preference in avoiding losses over acquiring equivalent gains. In the aforementioned scenario, at the beginning of the pandemic there was a lot to lose by missing out the vaccine, provided that the vaccine was effective. Correspondingly, the gains of not getting a vaccine to avoid ADR were not adequately relevant. The more doses were taken, however, the more this framework switched: if the vaccine was effective, there was something to gain from getting more doses, but if the vaccine was not safe, there was something to lose from each subsequent inoculation. In a nutshell, the advantages brought about by the vaccine turned out to be much less salient and conditioning than its possible disadvantages. Framing information in the most effective way, one that takes into account the loss-gain asymmetry, is therefore particularly important. During the pandemic, public immunization campaigns in most western countries focused much more on vaccine efficacy, rather than vaccine safety, despite contrary early indications [82]. If it could be argued that at the beginning of the campaign this could have been the correct strategy, it has lost its grip later on. Public communication should adapt not just the content of its messages, but also its focus, with the evolution of events. This should be regarded as an important insight for future vaccination campaigns.

### Internal and external reasons behind vaccination choice

Our exploration of the inner reasons behind the decision to vaccinate revealed results in line with our hypotheses and expectations. Whilst “COVID-19 related reasons” decreased in importance as the vaccination campaign was unfolding, the relevance of “social pressure reasons” progressively increased. Interestingly, if a decreased efficacy perception reasonably predicted a reduction in COVID-19 related reason and an increase in social ones, an increase of the safety during the third dose administration was associated with an increase of the influence of the social-related aspects, and a decrease of the impact of COVID-19 related aspects.

A possible explanation is that the concept of “vaccine safety” is certainly built upon the numerical threshold and data provided by researchers, but it could also be represented as a sort of socially constructed concept. For a layperson, efficacy can be gauged intuitively on a personal level by considering whether the disease is or is not caught after inoculation. On the contrary, judgment of safety (especially safety on medium/long term) is an inescapably statistical data. Therefore, a layperson has to rely much more on the “wisdom of the crowd” to intuitively gauge the safety of a vaccine. It follows that the process that leads to forming an opinion on how safe a vaccine is, is also the result of a process of social construction. That leads to the conclusion that with the increase of the importance of safety, we observe a corresponding increase in relevance of social-related aspects (see also [83]). Ergo, with a personal judgment of high safety comes the social expectation that others have the responsibility to vaccinate themselves, or at least to deem safety as high as one does. Consequently, COVID-19 related aspects become less relevant because there are other aspects that start playing a role in the equation.

### Trust, and its epistemic and intrinsic consequences

To encompass the plurality of potential causes of vaccine hesitancy, we have recently seen a shift in understating vaccine hesitancy from a model purely based on Information, called the Information- or Knowledge-Deficit Model, to a causation model that takes trust, and the lack of it, as a central aspect, around which an entire galaxy of intertwined questions pivots [71, 84, 85]. Moreover, a recently emerged consensus places lack of trust as one of, if not the, main factor behind vaccine hesitancy [86].

The most interesting result provided by a retrospective diachronic analysis of trust trends is not that trust decreases with the passage of time, but that trust in institutional agents decreases *more* than trust in the scientific community. This is yet another piece to consider, in light of the lively debate that has sparked over the question of what was more relevant in shaping compliance with medical policies between trust in public institutions or trust in science [87, 88]. This result can be explained in the light of two different aspects of trust, namely *epistemic* trust, and *intrinsic* trust (also known as reliability, see [65]). Placing high epistemic trust in someone means to consider its knowledge, competence, and capability well suited to treat a specific aspect. On the other hand, placing high intrinsic trust in someone means to consider its personal qualities, will, and determination well suited to treat a relevant aspect. Epistemic trust is a trust of the means, intrinsic trust is a trust of the ends.

Using this framework, public authorities are entrusted with intrinsic trust. To be considered trustworthy, they are expected to show characteristics of fairness, benevolence, and representation. On the contrary, the requirement placed on the scientific community places much more emphasis on the epistemic aspect of trust. To be trustworthy, they are required to be knowledgeable and competent. The events that unfolded during the pandemic decreased both epistemic trust, and intrinsic trust. Unquestionably, the pandemic saw some epistemic successes, such as the sharing of information amongst the community, and the response that was provided by most of the national healthcare services. These successes are not paired with an equal consideration of the work carried out by public authorities [89], given how their successes might not be ascribed (rightfully or not) to their intrinsic qualities. Therefore, the drop shown by our data reflects the different rates of reduction across different forms of trust, namely, the epistemic and the intrinsic.

## Limitations

In considering the findings of this research, it is important to also take into account the methodological limitations of this study. First, the cross-sectional study design poses a potential limitation, as participants were required to retrospectively recall decision-making processes related to vaccine acceptance. This introduces the risk of memory bias, with individuals selectively remembering and reporting information in alignment with current beliefs. Cognitive biases, such as carryover effects, may have impacted the accuracy of participant recollections. Another limitation concerns the experimental conditions. The absence of a pure control group without any vaccine-related message introduces confounding factors. Our control condition, indicating the unavailability of efficacy and safety information, may have influenced participants differently, affecting subsequent responses. Similarly, exclusively presenting positive information about vaccine efficacy and safety raises questions about generalizability. The absence of negatively framed messages limits insights into how individuals respond to information emphasizing potential risks associated with the vaccine. Lastly, the study’s sample may not be fully representative of the broader population. Despite efforts to balance demographic characteristics and align COVID-19 data with actual pandemic trends, inherent selection biases linked to online survey participation must be acknowledged. In summary, while the study contributes valuable insights into vaccine decision-making during the pandemic, it is crucial to recognize these limitations when interpreting the results.

## Conclusions

The perception of vaccines is a constantly evolving process, influenced by various dynamic factors such as perception of efficacy and safety, levels of trust, and individual motivations. Our study sheds light on the complex dynamics that underlie the perception of vaccine safety and efficacy, and their impact on willingness to vaccinate. We found that as the pandemic progressed, there was a decline in the perceived efficacy of the vaccine and a simultaneous rise in its perceived safety. Additionally, our findings showed that providing information about the safety of the vaccine had a noteworthy impact on influencing people’s willingness to get vaccinated, whereas information regarding effectiveness had an irrelevant or no influence. This suggests that the loss-gain asymmetry of prospect theory may be at play, with the avoidance of adverse effects being more salient than the benefits of protection.

These results have important implications for vaccination campaigns. They suggest that it may be necessary to adapt vaccination communication strategies throughout the pandemic, as the importance of vaccine safety and efficacy perceptions may vary over time. Overall, our study contributes to a better understanding of vaccine hesitancy and vaccine uptake and may inform the development of more effective vaccination campaigns and public health policies.

## Data Availability

The data that support the findings of this study are openly available in Open Science Framework database at https://osf.io/6efuz/?view_only=e876a0616cb54037b280546de350921e.

## References

[1] Carvalho T, Krammer F, Iwasaki A (2021). The first 12 months of COVID-19: a timeline of immunological insights. Nat Rev Immunol.

[2] Iezadi S, Gholipour K, Azami-Aghdash S, Ghiasi A, Rezapour A, Pourasghari H (2021). Effectiveness of non-pharmaceutical public health interventions against COVID-19: a systematic review and meta-analysis. PLoS ONE.

[3] Flaxman S, Mishra S, Gandy A, Unwin HJT, Mellan TA, Coupland H (2020). Estimating the effects of non-pharmaceutical interventions on COVID-19 in Europe. Nature.

[4] Perra N (2021). Non-pharmaceutical interventions during the COVID-19 pandemic: a review. Phys Rep.

[5] Shervani Z, Khan I, Khan T, Qazi UY (2020). COVID-19 vaccine. Adv Infect Dis.

[6] EU Vaccination Days [Internet]. Department for European Policies. [cited 2023 May 24]. http://www.politicheeuropee.gov.it/en/communication/news/european-vaccination-days-against-covid-19/

[7] Parino F, Zino L, Calafiore GC, Rizzo A. A model predictive control approach to optimally devise a two-dose vaccination rollout: a case study on COVID-19 in Italy. Int J Robust Nonlinear Control. 2021. 10.1002/rnc.5728 PMC8661761 34908815

[8] Bert F, Scaioli G, Vola L, Accortanzo D, Lo Moro G, Siliquini R (2022). Booster doses of anti COVID-19 vaccines: an overview of implementation policies among OECD and EU Countries. Int J Environ Res Public Health.

[9] Sallam M (2021). COVID-19 vaccine hesitancy worldwide: a concise systematic review of vaccine acceptance rates. Vaccines.

[10] Larson HJ (2022). Defining and measuring vaccine hesitancy. Nat Hum Behav.

[11] de Albuquerque Veloso Machado M, Roberts B, Wong BLH, van Kessel R, Mossialos E (2021). The relationship between the COVID-19 pandemic and vaccine hesitancy: a scoping review of literature until August 2021. Front Public Health.

[12] Paul KT, Zimmermann BM, Corsico P, Fiske A, Geiger S, Johnson S (2022). Anticipating hopes, fears and expectations towards COVID-19 vaccines: a qualitative interview study in seven European countries. SSM-Qual Res Health.

[13] Agranov M, Elliott M, Ortoleva P (2021). The importance of social norms against Strategic effects: the case of Covid-19 vaccine uptake. Econ Lett.

[14] Troiano G, Nardi A (2021). Vaccine hesitancy in the era of COVID-19. Public Health.

[15] Nguyen KH, Nguyen K, Corlin L, Allen JD, Chung M (2021). Changes in COVID-19 vaccination receipt and intention to vaccinate by socioeconomic characteristics and geographic area, United States, January 6–March 29, 2021. Ann Med.

[16] Lin C, Tu P, Beitsch LM (2020). Confidence and receptivity for COVID-19 vaccines: a rapid systematic review. Vaccines.

[17] Callaghan T, Moghtaderi A, Lueck JA, Hotez PJ, Strych U, Dor A et al. Correlates and disparities of COVID-19 vaccine hesitancy. Available SSRN 3667971. 2020.

[18] Kreps S, Prasad S, Brownstein JS, Hswen Y, Garibaldi BT, Zhang B, Kriner DL (2020). Factors associated with US adults’ likelihood of accepting COVID-19 vaccination. JAMA Netw open.

[19] Soares P, Rocha JV, Moniz M, Gama A, Laires PA, Pedro AR, Dias S, Leite A, Nunes C (2021). Factors associated with COVID-19 vaccine hesitancy. Vaccines.

[20] Caserotti M, Girardi P, Rubaltelli E, Tasso A, Lotto L, Gavaruzzi T (2021). Associations of COVID-19 risk perception with vaccine hesitancy over time for Italian residents. Soc Sci Med.

[21] Rubaltelli E, Tedaldi E, Orabona N, Scrimin S (2020). Environmental and psychological variables influencing reactions to the COVID-19 outbreak. Br J Health Psychol.

[22] Kennedy EB, Daoust JF, Vikse J, Nelson V (2021). Until I know it’s safe for me: the role of timing in COVID-19 vaccine decision-making and vaccine hesitancy. Vaccines.

[23] Robinson E, Jones A, Daly M (2021). International estimates of intended uptake and refusal of COVID-19 vaccines: a rapid systematic review and meta-analysis of large nationally representative samples. Vaccine.

[24] Ansani A, Marini M, Cecconi C, Dragoni D, Rinallo E, Poggi I, Mallia L (2022). Analyzing the perceived utility of Covid-19 countermeasures: the role of pronominalization, moral foundations, moral disengagement, fake news embracing, and health anxiety. Psychol Rep.

[25] Solís Arce JS, Warren SS, Meriggi NF, Scacco A, McMurry N, Voors M (2021). COVID-19 vaccine acceptance and hesitancy in low-and middle-income countries. Nat Med.

[26] Wouters OJ, Shadlen KC, Salcher-Konrad M, Pollard AJ, Larson HJ, Teerawattananon Y (2021). Challenges in ensuring global access to COVID-19 vaccines: production, affordability, allocation, and deployment. Lancet.

[27] Kaplan RM, Milstein A. Influence of a COVID-19 vaccine’s effectiveness and safety profile on vaccination acceptance. Proceedings of the National Academy of Sciences. 2021;118(10):e2021726118. 10.1073/pnas.2021726118 PMC7958192 33619178

[28] Davis CJ, Golding M, McKay R (2022). Efficacy information influences intention to take COVID-19 vaccine. Br J Health Psychol.

[29] Emu M, Chandrasekaran D, Mago V, Choudhury S. Validating optimal COVID-19 vaccine distribution models. In: Computational Science–ICCS. 2021: 21st International Conference, Krakow, Poland, June 16–18, 2021, Proceedings, Part I. Springer; 2021. pp. 352–66.

[30] Wang K, Wong ELY, Ho KF, Cheung AWL, Yau PSY, Dong D (2021). Change of willingness to accept COVID-19 vaccine and reasons of vaccine hesitancy of working people at different waves of local epidemic in Hong Kong, China: repeated cross-sectional surveys. Vaccines.

[31] Trabucco Aurilio M, Mennini FS, Gazzillo S, Massini L, Bolcato M, Feola A (2021). Intention to be vaccinated for COVID-19 among Italian nurses during the pandemic. Vaccines.

[32] Demichelis A, Marini M, Menicagli D, Mancini G, Bilancini E, Panizza F, Bellandi T, Boncinelli L, Galletti G, Caldesi R, Cevolani G. What leads to vaccine compliance? Evidence from healthcare workers in Italy. 10.1177/22799036251401950 PMC12966512 41798092

[33] Palma D, Hernández A, Picchio CA, Jodar G, Galbany-Estragués P, Simón P (2022). Confidence in a vaccine against COVID-19 among Registered nurses in Barcelona, Spain across two time periods. Vaccines.

[34] Folcarelli L, Miraglia del Giudice G, Corea F, Angelillo IF (2022). Intention to receive the COVID-19 vaccine booster dose in a university community in Italy. Vaccines.

[35] Gallant AJ, Nicholls LAB, Rasmussen S, Cogan N, Young D, Williams L (2021). Changes in attitudes to vaccination as a result of the COVID-19 pandemic: a longitudinal study of older adults in the UK. PLoS ONE.

[36] Zhu XM, Yan W, Sun J, Liu L, Zhao YM, Zheng YB (2022). Patterns and influencing factors of COVID-19 vaccination willingness among college students in China. Vaccine.

[37] Dai H, Saccardo S, Han MA, Roh L, Raja N, Vangala S (2021). Behavioural nudges increase COVID-19 vaccinations. Nature.

[38] Klüver H, Hartmann F, Humphreys M, Geissler F, Giesecke J (2021). Incentives can spur COVID-19 vaccination uptake. Proc Natl Acad Sci.

[39] Tentori K, Pighin S, Giovanazzi G, Grignolio A, Timberlake B, Ferro A. Default change nudges Covid-19 vaccine uptake: a randomized controlled trial. 2021. 10.1177/0272989X221101536 35658775

[40] Diament SM, Kaya A, Magenheim EB (2022). Frames that matter: increasing the willingness to get the Covid-19 vaccines. Soc Sci Med.

[41] Kahneman D, Tversky A. Prospect theory: An analysis of decision under risk. InHandbook of the fundamentals of financial decision making: Part I 2013 (pp. 99–127).

[42] Verma AA, Quinn KL, Detsky AS (2021). Marketing SARS-CoV-2 vaccines: an opportunity to test a Nobel Prize–winning theory. J Gen Intern Med.

[43] Huang Y, Liu W (2022). Promoting COVID-19 vaccination: the interplay of message framing, psychological uncertainty, and public agency as a message source. Sci Communication.

[44] Wang K, Wong EL, Cheung AW, Dong D, Yeoh EK (2023). Loss-framing of information and pre-vaccination consultation improve COVID-19 vaccine acceptance: a survey experiment. Front Public Health.

[45] Gong J, Zhang Y, Yang Z, Huang Y, Feng J, Zhang W (2013). The framing effect in medical decision-making: a review of the literature. Psychol Health Med.

[46] Peng L, Guo Y, Hu D (2021). Information framing effect on public’s intention to receive the COVID-19 vaccination in China. Vaccines.

[47] Ye W, Li Q, Yu S (2021). Persuasive effects of message framing and narrative format on promoting COVID-19 vaccination: a study on Chinese college students. Int J Environ Res Public Health.

[48] Gantiva C, Jiménez-Leal W, Urriago-Rayo J (2021). Framing messages to deal with the COVID-19 crisis: the role of loss/gain frames and content. Front Psychol.

[49] Gursoy D, Ekinci Y, Can AS, Murray JC (2022). Effectiveness of message framing in changing COVID-19 vaccination intentions: moderating role of travel desire. Tour Manag.

[50] Chen T, Dai M, Xia S, Zhou Y (2022). Do messages matter? Investigating the combined effects of framing, outcome uncertainty, and number format on COVID-19 vaccination attitudes and intention. Health Commun.

[51] Waterschoot J, Yzerbyt V, Soenens B, Van den Bergh O, Morbée S, Schmitz M et al. How do vaccination intentions change over time? The role of motivational growth. Health Psychol. 2022. 10.1037/hea0001228 36074600

[52] Del Riccio M, Boccalini S, Rigon L, Biamonte MA, Albora G, Giorgetti D (2021). Factors influencing SARS-CoV-2 vaccine acceptance and hesitancy in a population-based sample in Italy. Vaccines.

[53] Duong MC, Nguyen HT, Duong M, Evaluating (2022). COVID-19 vaccine hesitancy: a qualitative study from Vietnam. Diabetes Metab Syndr Clin Res Rev.

[54] Tu P, Kotarba M, Bier B, Clark R, Lin C (2022). Internal and external motivations and risk perception toward COVID-19 vaccination in adolescents in the US. Vaccines.

[55] Kraaijeveld SR (2020). Vaccinating for whom? Distinguishing between self-protective, paternalistic, altruistic and indirect vaccination. Public Health Ethics.

[56] Baeza-Rivera MJ, Salazar-Fernández C, Araneda-Leal L, Manríquez-Robles D (2021). To get vaccinated or not? Social psychological factors associated with vaccination intent for COVID-19. J Pac Rim Psychol.

[57] Allen JD, Mohllajee AP, Shelton RC, Othus MK, Fontenot HB, Hanna R (2009). Stage of adoption of the human papillomavirus vaccine among college women. Prev Med.

[58] Bell S, Clarke R, Mounier-Jack S, Walker JL, Paterson P (2020). Parents’ and guardians’ views on the acceptability of a future COVID-19 vaccine: a multi-methods study in England. Vaccine.

[59] Crawshaw J, Konnyu K, Castillo G, van Allen Z, Grimshaw J, Presseau J. Factors affecting COVID-19 vaccination acceptance and uptake among the general public: a living behavioural science evidence synthesis (v4, July 31st, 2021). Ott Ott Hosp Res Inst; 2021.

[60] Badur S, Ota M, Öztürk S, Adegbola R, Dutta A (2020). Vaccine confidence: the keys to restoring trust. Hum Vaccines Immunother.

[61] Rozek LS, Jones P, Menon A, Hicken A, Apsley S, King EJ (2021). Understanding vaccine hesitancy in the context of COVID-19: the role of trust and confidence in a seventeen-country survey. Int J Public Health.

[62] Siegrist M, Zingg A. The role of public trust during pandemics. Eur Psychol. 2014.

[63] Dubé E, MacDonald NE (2020). How can a global pandemic affect vaccine hesitancy?. Expert Rev Vaccines.

[64] Sapienza A, Falcone R (2022). The role of Trust in COVID-19 Vaccine Acceptance: considerations from a systematic review. Int J Environ Res Public Health.

[65] Wilholt T. Epistemic trust in science. Br J Philos Sci. 2013.

[66] Palamenghi L, Barello S, Boccia S, Graffigna G (2020). Mistrust in biomedical research and vaccine hesitancy: the forefront challenge in the battle against COVID-19 in Italy. Eur J Epidemiol.

[67] Falcone R, Colì E, Felletti S, Sapienza A, Castelfranchi C, Paglieri F. All we need is trust: how the COVID-19 outbreak reconfigured trust in Italian public institutions. Front Psychol. 2020;2585. 10.3389/fpsyg.2020.561747 PMC7562978 33132966

[68] Bengtsson R, Brommesson D (2022). Institutional trust and emergency preparedness: perceptions of Covid 19 crisis management in Sweden. J Contingencies Crisis Manag.

[69] Sudharsanan N, Favaretti C, Hachaturyan V, Bärnighausen T, Vandormael A (2022). Effects of side-effect risk framing strategies on COVID-19 vaccine intentions: a randomized controlled trial. Elife.

[70] Ahrendt D, Mascherini M, Nivakoski S, Sándor E. Living, working and COVID-19: Mental health and trust decline across EU as pandemic enters another year. Publications Office of the European Union; 2021.

[71] Falcone R, Ansani A, Colì E, Marini M, Sapienza A, Castelfranchi C (2022). Trusting COVID-19 vaccines as individual and social goal. Sci Rep.

[72] Ledda C, Costantino C, Liberti G, Rapisarda V (2022). The Italian version of the adult vaccine hesitancy scale (aVHS) for the Working-Age Population: cross-cultural adaptation, reliability, and Validity. Vaccines.

[73] George D, Mallery P. SPSS for Windows Step by Step: a simple study guide and reference, 17.0 update. Allyn & Bacon, Inc.; 2009.

[74] Kim HY (2013). Statistical notes for clinical researchers: assessing normal distribution (2) using skewness and kurtosis. Restor Dent Endod.

[75] Sadique Z, Edmunds WJ, Devlin N, Parkin D. Understanding individuals’ decisions about vaccination: a comparison between Expected Utility and Regret Theory models. 2005.

[76] Harris M, Hart J, Bhattacharya O, Russell FM (2023). Risk factors for SARS-CoV-2 infection during the early stages of the COVID-19 pandemic: a systematic literature review. Front Public Health.

[77] Dye TD, Barbosu M, Siddiqi S, Ramos JG, Murphy H, Alcántara L, Pressman E (2021). Science, healthcare system, and government effectiveness perception and COVID-19 vaccination acceptance and hesitancy in a global sample: an analytical cross-sectional analysis. BMJ open.

[78] Sun Y, Dai H, Wang P, Zhang X, Cui D, Huang Y, Zhang J, Xiang T (2022). Will people accept a third booster dose of the COVID-19 vaccine? A cross-sectional study in China. Front Public Health.

[79] Azim Majumder MA, Razzaque MS (2022). Repeated vaccination and ‘vaccine exhaustion’: relevance to the COVID-19 crisis. Expert Rev Vaccines.

[80] Sarkar J (2022). Do disease prevalence and severity drive COVID-19 vaccine demand?. Econ Anal Policy.

[81] Tversky A, Kahneman D (1981). The framing of decisions and the psychology of choice. Science.

[82] Ashworth M, Thunström L, Cherry TL, Newbold SC, Finnoff DC (2021). Emphasize personal health benefits to boost COVID-19 vaccination rates. Proc Natl Acad Sci.

[83] Hagger MS, Hamilton K (2022). Predicting COVID-19 booster vaccine intentions. Appl Psychol Health Well-Being.

[84] McClure CC, Cataldi JR, O’Leary ST (2017). Vaccine hesitancy: where we are and where we are going. Clin Ther.

[85] Goldenberg MJ (2016). Public misunderstanding of science? Reframing the problem of vaccine hesitancy. Perspect Sci.

[86] Goldenberg MJ. Vaccine hesitancy: public trust, expertise, and the war on science. University of Pittsburgh; 2021.

[87] Bicchieri C, Fatas E, Aldama A, Casas A, Deshpande I, Lauro M (2021). In science we (should) trust: expectations and compliance across nine countries during the COVID-19 pandemic. PLoS ONE.

[88] Sulik J, Deroy O, Dezecache G, Newson M, Zhao Y, El Zein M et al. Facing the pandemic with trust in science. Humanit Soc Sci Commun. 2021;8(1).

[89] Barattucci M, Pagliaro S, Ballone C, Teresi M, Consoli C, Garofalo A et al. Trust in science as a possible mediator between different antecedents and COVID-19 booster vaccination intention: an integration of health belief model (HBM) and theory of planned behavior (TPB). Vaccines. 2022;10(7):1099.World Health Organization (WHO). Summary WHO SAGE conclusions and recommendations on Vaccine Hesitancy. 10.3390/vaccines10071099 PMC9320855 35891265

